# Mutations in the maize zeta-carotene desaturase gene lead to viviparous kernel

**DOI:** 10.1371/journal.pone.0174270

**Published:** 2017-03-24

**Authors:** Yan Chen, Jiankun Li, Kaijian Fan, Yicong Du, Zhenjing Ren, Jing Xu, Jun Zheng, Yunjun Liu, Junjie Fu, Dongtao Ren, Guoying Wang

**Affiliations:** 1 Institute of Crop Sciences, Chinese Academy of Agricultural Sciences, Beijing, China; 2 College of Biological Sciences, China Agricultural University, Beijing, China; 3 National Maize Improvement Center of China, Beijing Key Laboratory of Crop Genetic Improvement, China Agricultural University, Beijing, China; University of Manitoba, CANADA

## Abstract

Preharvest sprouting reduces the maize quality and causes a significant yield loss in maize production. *vp-wl2* is a *Mutator* (*Mu*)-induced viviparous mutant in maize, causing white or pale yellow kernels, dramatically reduced carotenoid and ABA content, and a high level of zeta-carotene accumulation. Here, we reported the cloning of the *vp-wl2* gene using a modified digestion-ligation-amplification method (DLA). The results showed that an insertion of *Mu9* in the first intron of the zeta-carotene desaturase (*ZDS*) gene results in the *vp-wl2* mutation. Previous studies have suggested that *ZDS* is likely the structural gene of the *viviparous9* (*vp9*) locus. Therefore, we performed an allelic test using *vp-wl2* and three *vp9* mutants. The results showed that *vp-wl2* is a novel allele of the *vp9* locus. In addition, the sequences of *ZDS* gene were identified in these three *vp9* alleles. The *vp-wl2* mutant gene was subsequently introgressed into four maize inbred lines, and a viviparous phenotype was observed with yield losses from 7.69% to 13.33%.

## Introduction

Maize (*Zea mays* L.) is one of the major cereal crops in China and many countries. Preharvest sprouting is also known as vivipary, which has been described since the early 1920s [1–5]. To our knowledge, approximately fifteen viviparous mutants have been identified. According to the criteria proposed by Robertson, the viviparous mutants have been classified into two classes [6]: Class One, in which mutant genes, including *vp1/vp4*, *vp6*, *vp8*, *vp10/vp13*, *vp14*, and *vp15*, do not affect the endosperm and seedling color; and Class Two, in which mutant genes, including *vp2*, *vp5*, *vp7/ps1*, *vp9*, *vp12/lw2*, *y9*, *w3*, and *rea1*, will alter carotenoid and chlorophyll synthesis. Until recently, eight viviparous loci have been cloned, and further studies are needed to examine other mutants. Maize *vp1/vp4* mutant showed reduced sensitivity to ABA and blocked anthocyanin synthesis in aleurone and embryo tissues. The *vp1* locus was cloned using transposon tagging, and subsequent experiments have shown that the VP1 protein may control the anthocyanin pathway by regulating the *C1* gene [7]. VP1 can complement the Arabidopsis *abi3* mutant allele, as it mediates a novel interaction between ABA and auxin signaling [8]. The *vp8* gene increases the number of vegetative phytomers and delays the juvenile-to-adult transition [9]; molecular analysis revealed that *Vp8* encodes the putative peptidase ALTERED MERISTEM PROGRAM1 (AMP1), which is required for the synthesis of an unidentified signal that integrates meristem and embryo formation in seeds [10]. Another allele of *vp8*, *d2003*, contains a premature stop codon in the *Vp8* gene, resulting in a dwarf phenotype in maize plants [11]. A transposon-tagged allele of *vp10* was cloned in a previous study, and the results showed that *Vp10* encodes the ortholog of *Cnx1*, which catalyzes the final common step of MoCo synthesis [12]. The synthesis of ABA, auxin and nitrate depends on MoCo; thus, mutations in *Cnx1* (*vp10*) will result in a viviparous phenotype. Maize *vp14* was cloned using transposon tagging, and the results of subsequent experiments suggested that the VP14 protein sequence was similar to that of bacterial lignostilbene dioxygenases (LSD), which catalyze a double bond cleavage reaction [13]. VP14 protein catalyzes the cleavage of 9-*cis*-epoxy- carotenoids to form C_25_ apo-aldehydes and xanthoxin, a precursor of ABA in higher plants [14,15]. The molecular analysis of mutations confirmed that *Vp15* encodes the molybdopterin synthase (ZmCNX7) [16]. The *vp15* mutant showed the reduced activities of several enzymes and ABA biosynthesis requiring MoCo. The cDNA encoding PDS has been isolated and characterized, showing that this enzyme catalyzes two desaturation steps converting phytoene to zeta-carotene [17]. The transcript of the *PDS* gene in the *vp5* mutant contains multiple insertions and deletions, and the 55-kDa PDS protein is not expressed in the *vp5* mutant; thus, the causal gene of *vp5* must be *Pds* [18]. The *ps1/vp7* locus was cloned using an *Ac*-based gene tagging approach. The *Ps1* gene is predicted to encode lycopene β-cyclase [19], which affects ABA synthesis resulting in a viviparous phenotype. The maize *y9* mutant has a slightly viviparous phenotype, and *Y9* encodes a factor required for isomerase activity upstream of CRTISO, referred to as Z-ISO, and it has an activity that catalyzes the cis- to trans-conversion of the 15-cis-bond in 9, 15, 9’-tri-cis-zeta-carotene, the substrate of ZDS [20]. Apart from these typical viviparous mutants, the *naked endosperm* (*nkd*) mutants, which produce multiple outer cell layers, are prone to vivipary [21]. Luo and Wurtzel [22] isolated the cDNA of maize *ZDS* in 1999 and subsequently mapped this gene to the maize 7S chromosome. Previous studies have suggested that *vp9* is a candidate locus for this structural gene, as expression of the *ZDS* transcript is reduced in the *vp9-Mum* mutant. However, it is also possible that *ZDS* transcription be regulated by the VP9 protein [23]. Apart from the cloned vivipary loci described above, the following loci have not been cloned. The *vp2* locus is mapped to bin 5.03,whereas the *vp12/lw2* locus is located on the long arm of chromosome 6 [24], the *w3* locus mapped to bin 2.06, the *rea1* locus mapped to bin 3.05, and there are no details concerning the *vp6* locus. The *dek33* mutant is a kernel-defect mutant mapped to bin 5.04, and occasionally, this mutant shows a viviparous phenotype.

In the present study, a novel viviparous mutant, belonging to the Class Two viviparous mutants, was identified in the progeny of *Mutator* (*Mu*) active lines, and this mutant was named as *vp-wl2*. We cloned the causal gene of the *vp-wl2* mutant, which encodes zeta-carotene desaturase (ZDS) protein. Allelic tests showed that *vp-wl2* and three *vp9* mutants were functional alleles and confirmed that the mutation of the *ZDS* gene will lead to viviparous kernels. The subcellular localization analysis revealed that ZDS is localized in chloroplasts of maize protoplasts. These results indicated that *ZDS* is the causal gene of the *vp9* locus. Moreover, ZDS is targeted in chloroplasts and participates in carotenoid biosynthesis.

## Materials and methods

### Maize materials

The maize *vp-wl2* mutant was discovered in a selfing population derived from the hybridization of *Mutator* (*Mu*) active lines with B73. The *vp-wl2* was introgressed into 4 maize inbred lines, namely Chang7-2, Zheng58, Mo17, and B73, by several cycles of backcrossing. Three maize *vp9* mutants, vp9-R (707E), vp9-99-2226-1 (721I), and vp9-8113 (722A), were obtained from the Maize Genetics Cooperation Stock Center. All the lines were cultivated under field conditions twice a year in Beijing and Sanya, respectively.

### Analysis of carotenoids and ABA content

To analyze the carotenoids of maize endosperm, a YMC-Pack C30 reverse-phase chromatographic column (250 × 4.6 mm, 5 μm; YMC Europe) was used in an HPLC system. The carotenoid extraction and analysis was performed according to a previous study [25–27]. One gram of maize endosperm was harvested at 15 days after pollination (DAP) and ground in liquid nitrogen under yellow light condition; the following extraction process was performed under this condition, and 6 mL of extraction buffer (ethanol containing 1% butylated hydroxy toluene) was then added and incubated at 85°C for 6 min, followed by the addition of 120 μL of KOH (1g/mL) and further incubation at 85°C for 10 min. The samples were vortexed twice during the incubations. After saponification, the samples were cooled on ice, and an additional 4 mL of pre-cooled water was added, followed by the addition of 3 mL of 2:1 petroleum ether:diethyl ether (v/v), vortexing, and centrifugation at 2700 rpm for 5 min. The supernatant was transferred to a new tube. The extraction step was repeated three times, and the supernatant was collected in the same tube, followed by the addition of 4 mL of pre-cooled water to wash the supernatant and centrifugation at 2700 rpm for 5 min. Then, the supernatant was transferred to a 10-mL glass tube and vacuum dried. The extracted carotenoids were dissolved in 1000 μL of the mobile phase for HPLC analysis. Synthetic standards (Lutein, Zeaxanthin, β-cryptoxanthin, β-carotene, α-carotene, and zeta-carotene) were used in the HPLC analysis. The ABA content was measured on a Plant Hormone Platform at the Institute of Genetics and Developmental Biology, CAS.

### Cloning of the *vp-wl2* gene

The *vp-wl2* gene was cloned using the digestion-ligation-amplification (DLA) method [28], with some modifications. In the *vp-wl2* population, ear lines were selected, comprising five individuals with non-segregated ears (wild-type) and seven individuals with segregated ears (heterozygous). The genomic DNA was extracted using the CTAB method. A total of 2 μg of genomic DNA was digested using the restriction enzyme NspI (New England Biolabs (Beijing) LTD), and the adaptor, 5’-CAGAACGTCACAGCATGTCATG-3’, was ligated to the digested DNA fragments using T4 DNA ligase (New England Biolabs (Beijing) LTD). The DNA fragments were subsequently purified using the TIANquick Midi Purification Kit (TIANGEN BIOTECH (BEIJING) CO., LTD), and the purified products were used as templates for the first-round of PCR using the following cycling conditions: initial denaturation at 94°C for 5 min, followed by 30 cycles of denaturation at 94°C for 30 s, primer annealing at 60°C for 30 s, and extension at 72°C for150 s, with a final extension at 72°C for 7 min. The products of the first-round PCR were used as the templates for the second-round of PCR, using the same PCR program as used for the first-round of PCR, except that 35, not 30, cycles were used. All the primers are listed in S1 Table. The second-round PCR products were separated on 6% polyacrylamide gels, and the targeted bands were collected and purified using the Poly-Gel DNA Extraction Kit (OMEGA BIO-TEK). The targeted DNA fragments were cloned into the *pEASY*-T1 Cloning Vector and subsequently transformed into Trans5α Chemically Competent Cells (the vector and host cells were obtained from TransGen Biotech LTD). The clones were sequenced using the universal primers *M13F* and *M13R*, and the results were blasted in the Maize GDB (http://www.maizegdb.org) and NCBI (http://www.ncbi.nlm.nih.gov) databases to identify the *Mu* insertion and its flanking sequence.

### Validation and expression analysis of *the vp-wl2* mutant

According to the procedure described above, we identified a *Mu9* insertion and its flanking sequence, and gene- and *Mu*-specific primers were designed to validate the one-to-one correspondence of the genotype with the phenotype in a larger population. The primers are listed in S1 Table (primer validation pairs 1 and 2). These two primer pairs were used to identify the three genotypes (*AA*, *Aa*, and *aa*). Normal kernels from the heterozygous ears were planted in the winter of 2013 in Sanya, and approximately three hundred individual genotypes were identified using primer validation pairs 1 and 2. All individuals were self-pollinated to identify the phenotypes, and the population was used to validate the relationship between genotype and phenotype.

The normal kernels and some white but dormant kernels were planted in a greenhouse; the seedlings were harvested to extract genomic DNA using the CTAB method. Total RNA was extracted using the RNAprep Pure Plant Kit (TIANGEN BIOTECH (BEIJING) CO., LTD). The genomic DNA was used to identify the genotype of each plant, and 1μg of total RNA was used to generate first-strand cDNA using reverse transcriptase. The gene expression levels were measured using qPCR and RT-PCR, with three specific primer pairs in the *ZDS* gene, and the *GAPDH* gene was used as an internal control. All primers are listed in S1 Table (RT-PCR primer pairs).

### Allelic test between *vp-wl2* and *vp9*

Normal kernels in the heterozygous ears of *vp-wl2* and the three *vp9* maize lines (vp9-R (707E), vp9-99-2226-1 (721I), and vp9-8113 (722A)) were planted in Beijing and Sanya in 2014 and 2015. The genotype of each plant in the *vp-wl2* population was determined. The heterozygous plants were crossed with each *vp9* line, but the *vp9* lines were self-pollinated to identify the heterozygous individuals.

### Identifying the *ZDS* gene sequences and transcript abundances in three *vp9* mutants

ZUTR-1F, ZUTR-1R, and twelve other sequencing primers (S1 Table) were used to identify the genomic and CDS sequences of three *vp9* alleles. Viviparous and normal kernels were collected from ears 25 DAP, and the total RNA and genomic DNA were extracted. ZUTR-1F and ZUTR-1R were used to amplify the cDNA and genomic DNA of each sample, the high fidelity enzyme KOD FX (TOYOBO) was used, and all products were sequenced. The primers spanning the exons of *ZDS* were designed to amplify the genomic DNA of vp9-99-2226-1, and all primers are listed in S1 Table. Three nested primers, SP1, SP2, and SP3, in the *ZDS* gene were designed, and thermal asymmetric interlaced PCR was used to obtain the 5’ flanking sequence of the *ZDS* gene in the vp9-99-2226-1 mutant. Thermal asymmetric interlaced PCR was performed using a Genome Walking Kit (TaKaRa D316). qPCR was performed using a specific primer pair (ZUTR-3F and ZUTR-3R) of *ZDS*, and *GAPDH* served as an internal control. Three technical replicates were conducted for each sample.

### Introgress the *vp-wl2* mutant gene into other backgrounds and yield evaluation

To identify phenotypes of the *vp-wl2* mutant gene in other backgrounds, this mutant was introgressed into four maize inbred lines, namely Chang7-2, Zheng58, Mo17, and B73. The heterozygous individuals carrying the *vp-wl2* mutant gene were crossed with the four maize inbred lines, and markers were used to determine the genotype of the F_2_ population. All the individuals were self-pollinated. Mature ears were harvested and dried under the same conditions. Normal kernels and viviparous kernels from the same ear were separated. The 100-kernel weight was measured between normal and viviparous kernels to determine the yield loss ratio.

### Subcellular localization of ZDS protein

The CDS sequence of the *ZDS* gene from maize inbred line B73 was amplified, and the T base pair in the stop codon was removed. The modified CDS was cloned into an entry vector (pGWC), and an LR reaction was performed between the pGWC-*ZDS* vector and the pEarleyGate 101 vector. LR Clonase^®^ II Plus was obtained from Invitrogen^™^. The *ZDS* gene was cloned into the pEarleyGate 101 vector, overexpressing the fused ZDS-YFP protein. A chemical method was used to transform the vector into the *Agrobacterium tumefaciens* strain GV3101. Maize protoplasts were used to perform the transient expression of fused ZDS-YFP protein. Etiolated maize seedlings of the inbred line B73 were cultivated under dark conditions for ten days, and the leaves were harvested to isolate the protoplasts. A PEG-calcium solution was used to perform the transfection using a method previously described [29–31]. Following transfection, the maize protoplasts were suspended in W5 buffer and incubated at room temperature in the dark for fourteen hours. The transient expression of ZDS-YFP fusion protein was observed using a confocal laser-scanning fluorescence system (Zeiss LSM700). *Nicotiana benthamiana* was planted in a greenhouse for 40 days. The transformed *Agrobacterium tumefaciens* strain GV3101 was cultured in liquid YEB medium containing acetosyringone and magnesium chloride; the cells were collected and resuspended for injection into the leaves of *Nicotiana benthamiana*. The injected plants were maintained in the dark for 24 hours and transferred to light for 48 hours. The leaves were used to observe the transient expression of fused ZDS-YFP protein using a Zeiss LSM700 system.

## Results

### Characterization of a novel *Mu*-tagged viviparous mutant *vp-wl2*

**Fig 1:**
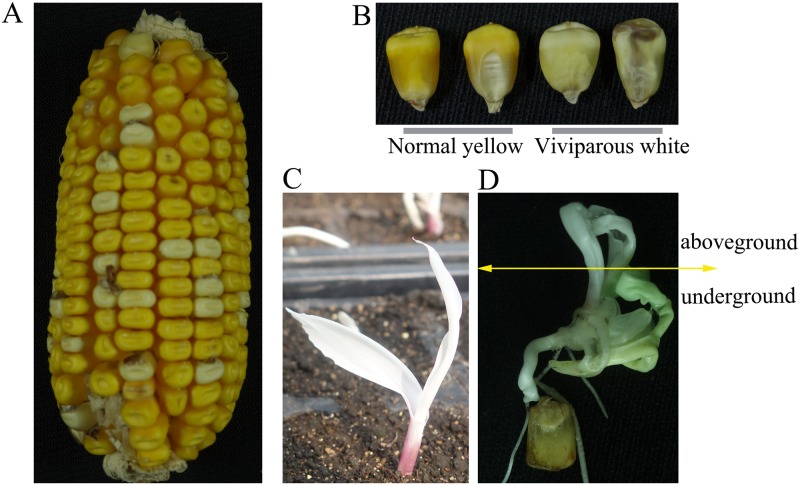
Phenotypes of the *vp-wl2* mutant. (A) Heterozygous ear of the *vp-wl2* mutant.(B) Normal and viviparous kernels from the same ear of the *vp-wl2* mutant.(C) Albino seedling of a white mutant seed, which was dormant and did not sprout on the ear. (D) An albino seedling, for which the seedling above the ground is white, and the seedling under the ground is faint blue-green.

A novel maize viviparous mutant, *vp-wl2*, was selected among the progeny of *Mu*-tagged population. In heterozygous ears (Fig 1A and 1B), some kernels appear white or pale yellow, and germinate before harvest. Occasionally, some white but dormant seeds were observed in the heterozygous ears. The yellow normal and white mutant kernels were counted on the heterozygous ears. A fitness test was performed, and the segregation ratio was 3:1 (S2 Table). Similar to other maize endosperm mutants, a hetero-fertilization phenomenon [32–34] was observed for the *vp-wl2* mutant kernels. A total of 96 white but dormant seeds were collected and planted in the greenhouse. Most of the seedlings were white (Fig 1C and S1 Fig). However, there were six green seedlings, indicating that the six white but dormant seeds have different genotypes between endosperm and embryo. Interestingly, a faint blue-green pigment was observed for mutants grown under low light conditions, i.e., ears wrapped in husk leaves or buried in the soil (Fig 1D and S2 Fig).

### ABA content and carotenoid profiles of *vp-wl2*

The endosperms and embryos of *vp-wl2* mutants and normal seeds at 15 DAP were dissected for the determination of ABA content. The results showed that the ABA content in the endosperms of *vp-wl2* mutants had no significant difference from the normal ones. However, the ABA content in the embryos of *vp-wl2* mutants was dramatically lower than that in the normal embryos (Table 1).

**Table 1:** ABA content of *vp-wl2* mutant and normal kernels.

Samples	Tissue	ABA pg/mg FW				
		I	II	III	Average	SD
*vp-wl2*_B73	endosperm	7.11	7.32	7.37	7.27	0.14
WT_B73	endosperm	8.07	7.91	7.90	7.96	0.10
*vp-wl2*_B73	embryo	9.11	na	na	9.11	na
WT_B73	embryo	52.51	na	na	52.51	na
*vp-wl2*_Mo17	embryo	4.14	na	na	4.14	na
WT_Mo17	embryo	21.56	na	na	21.56	na

FW, fresh weight; *vp-wl2* and WT represent mutant kernels and normal kernels, respectively; na, no data available; SD represents the standard deviation, and the symbol I, II, and III represent three repeats.

**Fig 2:**
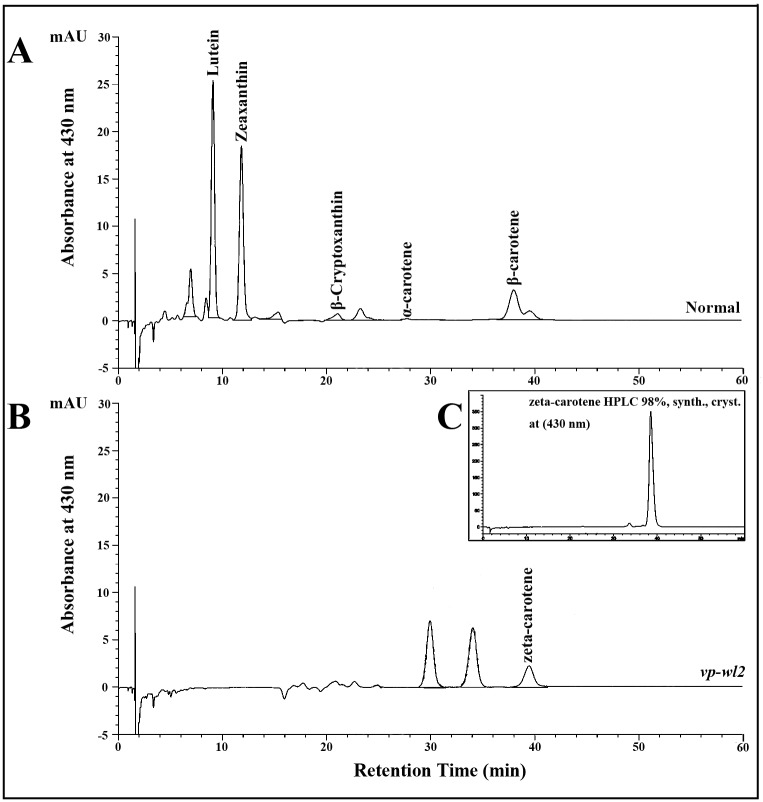
HPLC chromatograms of carotenoids in normal and *vp-wl2* mutant endosperms. (A) HPLC chromatograms of normal endosperms recorded at 430 nm. (B) HPLC chromatograms of *vp-wl2* mutant endosperms recorded at 430 nm. (C) HPLC chromatograms of synthetic zeta-carotene recorded at 430 nm.

High-performance liquid chromatography (HPLC) was used to analyze the carotenoid profiles in *vp-wl2* mutant and normal endosperms. As shown in Fig 2, the composition of the carotenoids showed a large difference between *vp-wl2* mutant and normal seeds. For the *vp-wl2* mutants, there are three distinct absorption peaks (Fig 2B). The absorption peak appearing near 40 min was zeta-carotene according to the synthetic zeta-carotene standards (Fig 2C). However, the absorption peaks of lutein, zeaxanthin, β-cryptoxanthin, and β-carotene in normal seeds (Fig 2A) were undetectable in *vp-wl2* mutants. Nevertheless, the *vp-wl2* mutant accumulated zeta-carotene and was unable to produce the necessary carotenoids and xanthophylls, which leads to the deficiency of ABA.

### Cloning of the *vp-wl2* gene

**Fig 3:**
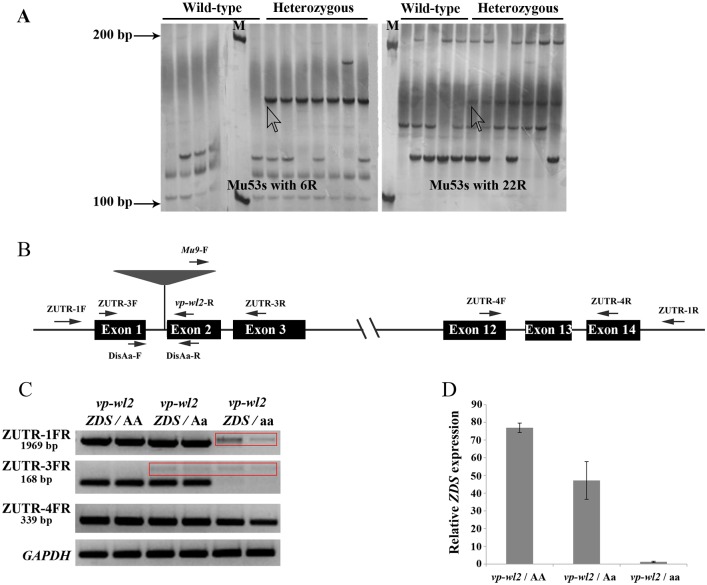
Cloning and molecular characterization of the *ZDS* gene. (A) Polyacrylamide gel electrophoresis results of DLA cloning, in which wild-type represents five individuals with non-segregated ears, and heterozygous represents seven individuals with segregated ears. (B) The *ZDS* gene profile and position of the primers. (C, D) Analysis of the *ZDS* gene expression level in the *vp-wl2* population using RT-PCR (C) and qPCR (D).

Modified DLA method was adopted to obtain the *Mu* insertion and its flanking sequence. The genomic DNA of five wild-type individuals and seven heterozygous individuals were extracted by the CTAB method and digested by restriction enzyme NspI, and an oligonucleotide adaptor was ligated to the digested DNA fragments. After two rounds of PCR, the two primer pairs (Mu53s with 6R and Mu53s with 22R) produced two specific bands in the seven heterozygous individuals but not in the five wild-type individuals (Fig 3A). The DNA in bands was isolated and sequenced. Only a base pair difference was observed between the two sequences (S3 Fig). The sequence was blasted in NCBI and MaizeGDB, showing that this DNA fragment contained a partial sequence of *Mu9* and a partial sequence of the zeta-carotene desaturase gene (*ZDS*, GRMZM2G454952). The profile of the *ZDS* gene and position of each primer are displayed in Fig 3B. The *ZDS* gene contained 14 exons and 13 introns, the *vp-wl2* mutant had an insertion of *Mu9* in the first intron, which disrupted the normal splicing of *ZDS*. Primer validation pairs 1 and 2 (*Mu9*-F with *vp-wl2*-R and DisAa-F with DisAa-R) were designed to identify the insertion and the genotype. Approximately three hundred individuals were genotyped, and co-segregation was observed between genotype and phenotype.

Three primer pairs were used to perform the RT-PCR analysis, and the amplified region is indicated in Fig 3B, in which a primer pair spanning the insertion of *Mu9* (ZUTR-3F with ZUTR-3R) was also used to perform qPCR. The results showed that the full-length CDS of the *ZDS* gene was not detected in the *vp-wl2* mutant, whereas a larger but blurred band, indicated in the red box (Fig 3C), was amplified. Another primer pair, ZUTR-3F with ZUTR-3R, showed that the *ZDS* gene was not expressed in the *vp-wl2* mutant; however, a larger but blurred band was also detected in the *vp-wl2* mutant and the heterozygous individuals. The bands in the red box were collected, purified and subsequently cloned into the *pEASY*-Blunt Cloning Vector, followed by sequencing. The results showed that the insertion of *Mu9* in the first intron affects the splicing of pre-mRNA (S4 Fig), producing larger but abnormal transcripts. These transcripts contained partial sequences of *Mu9* and some intron sequences of *ZDS*. The sequences of these abnormal transcripts, adjacent to the 3’ end of the gene were precisely the same as those in normal individuals. Thus, the amplicon resulting from primer pair ZUTR-4F and ZUTR-4R can be detected in the *vp-wl2* mutant, whereas the expression level in the mutant allele was lower than that in normal seedlings (Fig 3C). The results of qPCR showed that the *ZDS* gene expression was normal in wild-type plants; however, the expression of the *ZDS* gene could not be detected in the *vp-wl2* mutant, and compared with wild-type plants, the *ZDS* gene expression level was dramatically reduced in heterozygous plants (Fig 3D).

### *vp-wl2* is allelic to *vp9*

In 1999, Luo and Wurtzel [22] isolated the cDNA of maize *ZDS* gene and mapped this gene to maize chromosome 7S. Moreover, *vp9* was suggested as a candidate locus for the *ZDS* gene [23]. In order to confirm this suggestion, the *ZDS* mutant (*vp-wl2*) was used in an allelic test with *vp9*.

The heterozygous plants of three maize *vp9* mutants (vp9-R (707E), vp9-99-2226-1 (721I), and vp9-8113 (722A)) were crossed with the heterozygous plants of *vp-wl2*, producing ears with 3/4 normal and 1/4 viviparous kernels (Table 2). The allelic test results indicated that *vp-wl2* is a novel allele of *vp9* and confirmed that the structural gene of the *vp9* locus is *ZDS*, which encodes a zeta-carotene desaturase protein that functions in the carotenoid biosynthetic pathway (S5 Fig).

**Table 2:** Segregation analysis of the allelic test ears using a fitness test.

Cross combination of heterozygous individuals	Normal kernels	Viviparous kernels	χ^2^ value	3.84 (α = 0.05)	6.63 (α = 0.01)
*vp-wl2* × vp9-R	2697	883	0.20	ns	ns
*vp-wl2* × vp9-99-2226-1	1644	540	0.07	ns	ns
*vp-wl2 ×* vp9-8113	3145	997	1.86	ns	ns

"ns" means not significant.

### Analysis of the *ZDS* gene sequence and its expression levels in three *vp9* alleles

**Fig 4:**
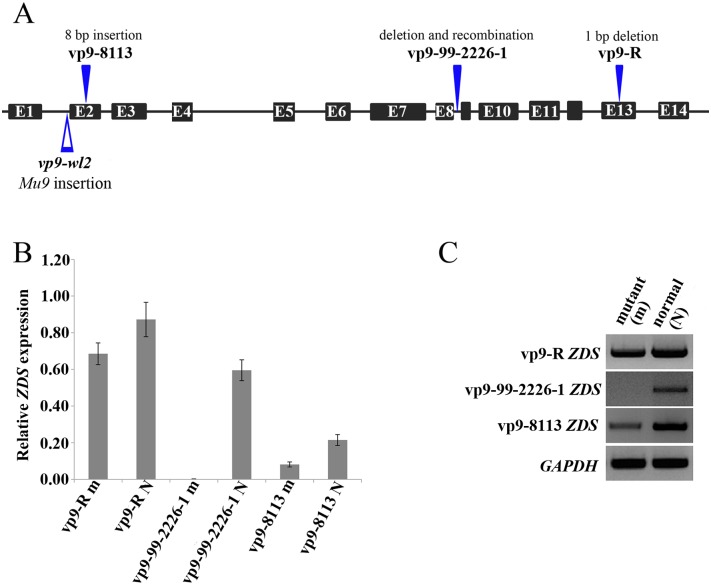
Three *vp9* lines and their *ZDS* mutant alleles. (A) Profile of the *ZDS* gene mutation in the three *vp9* lines. (B, C) The *ZDS* gene transcript abundance analysis using RT-PCR and qPCR, in which “N” represents normal kernels, and “m” represents viviparous kernels.

**Fig 5:**
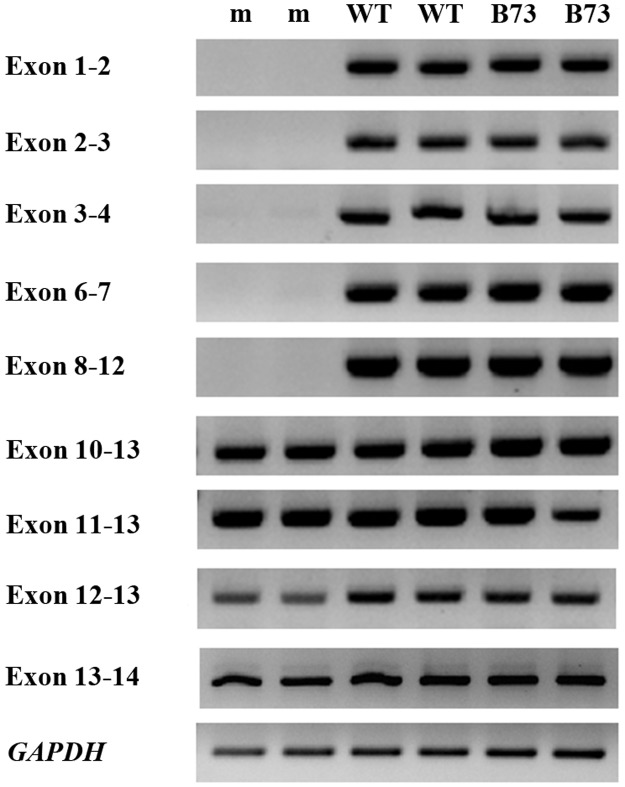
Amplification of the *ZDS* gene fragments in vp9-99-2226-1. Two repetitions were performed, in which “m” represents vp9-99-2226-1 mutant, “WT” represents its wild-type counterpart, and “B73” represents the maize inbred line B73. Exons 1–2 represent the PCR products spanning the first and the second exons; the other names are indicated in the same manner, and *GAPDH* served as an internal control gene.

RT-PCR was used to obtain the CDS sequences of the *ZDS* gene from three *vp9* lines. Compared with its wild-type individuals, vp9-R contained an 8-bp deletion in the 5’ UTR of *ZDS*, and a 1-bp deletion in the coding region, causing a frameshift mutation; and it also has 19 single base mutations (Fig 4A). The second *vp9* allele, vp9-8113, contained an 8-bp insertion in the coding region of ZDS protein, causing a frameshift mutation (Fig 4A). The third *vp9* allele, vp9-99-2226-1, had a large fragment deletion in the *ZDS* gene region; fragments from exon 1 to exon 8 cannot be amplified from the genomic DNA, which indicated that this fragment does not exist in the mutant genome (Fig 5). Thermal asymmetric interlaced PCR was performed based on three nested primers in *ZDS*, and a flanking sequence, approximately 1500 bp, was obtained in the vp9-99-2226-1 mutant; this fragment cannot be fully mapped to B73 reference genome (http://www.maizegdb.org/), but it has high similarity with a mobile element in Mo17 genome, and the identity is 92.0%.

Relative *ZDS* expression was measured by qPCR (Fig 4B). The expression level of *ZDS* in the vp9-R mutant was slightly reduced compared with its counterpart; however, the expression level of *ZDS* was greatly reduced in the vp9-8113 mutant, and the *ZDS* transcript cannot be detected in the vp9-99-2226-1 mutant.

### The *vp-wl2* mutant gene leads to viviparous kernels and the yield loss in various backgrounds

To validate whether the *vp-wl2* mutant gene works in other background or not, it was introgressed into four widely used maize inbred lines (B73, Mo17, Chang7-2, and Zheng58). Under the background of four maize inbred lines, the F_2_ ears showed the segregation of normal kernels and viviparous kernels, which indicated that the *vp-wl2* mutant gene abolished the normal function of ZDS protein in these four maize lines.

**Fig 6:**
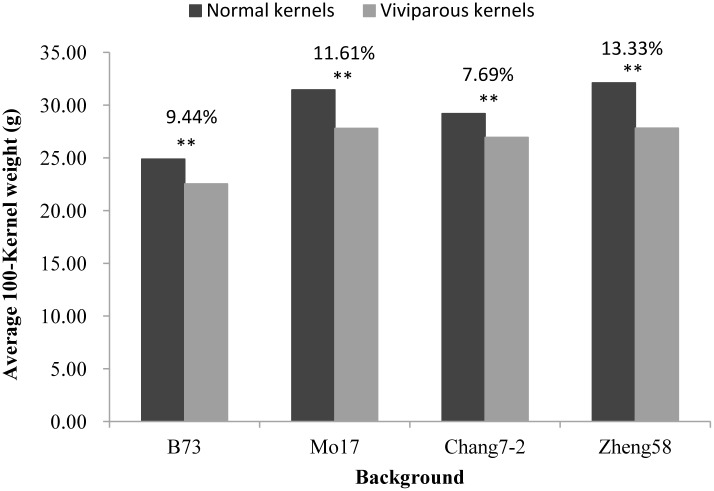
Histogram of the 100-kernel weight between normal and viviparous kernels. The x-axis represents four maize backgrounds, B73, Mo17, Chang7-2, and Zheng58; the y-axis represents average 100-kernel weight, and the percentage indicated above represents the degree of yield decrease.

The results also showed that the 100-kernel weight of viviparous kernels was significantly lower than that of normal kernels, which caused a yield loss from 7.69% to 13.33% in the four maize backgrounds (Fig 6).

### ZDS protein is targeted to chloroplasts

**Fig 7:**
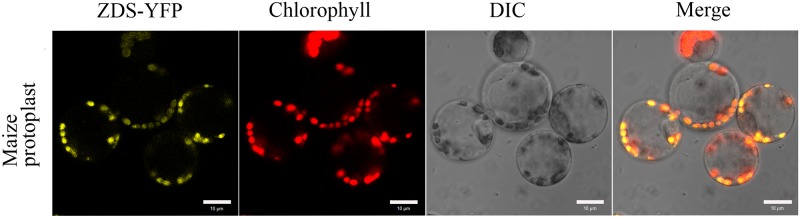
Subcellular localization of ZDS protein. The ZDS protein was fused with yellow fluorescence protein (YFP), and transiently expressed in maize protoplasts, red fluorescence was automatically emitted by chloroplasts. Bar = 10 μm.

The carotenoid biosynthetic pathway is summarized in S5 Fig; ZDS protein catalyzed a two-step desaturation from 9,9’-dis-cis-zeta-carotene to 7’,9’-cis-lycopene [20,35]. To identify the subcellular localization of ZDS protein, it was fused with yellow fluorescence protein (ZDS-YFP) and transiently expressed in maize protoplasts and tobacco leaves. In maize protoplast, some spots around the cell emitted yellow fluorescence; they can overlap with red chlorophyll auto-fluorescence, which indicated that ZDS protein localized in chloroplasts (Fig 7). In tobacco leaves, this fused ZDS-YFP protein also localized in chloroplasts (S6 Fig). In S6 Fig, a piece of tobacco leaf, which contained mesophyll cells and epidermal cells, and an epidermal cell were observed under the confocal laser microscopy. Many yellow punctate spots in different shapes existed in epidermal cells, which emitted bright yellow fluorescence, while the red fluorescence of chloroplasts is dim (marked with white arrows in S6 Fig), so the merged pictures did not show orange fluorescence. However, in the mesophyll cells many chloroplasts emitted red fluorescence, and they can overlap with yellow fluorescence, emitting orange fluorescence. These results suggested that the maize ZDS protein was also targeted to chloroplasts in tobacco.

## Discussion

Carotenoids are the second most abundant pigments in nature, and the carotenoid family comprises more than 700 members [36]. In higher plants, carotenoids are primarily synthesized in chloroplasts by nuclear-encoded enzymes [37], and some carotenoids are synthesized in plastids, such as corolla, radical and nectarial chromoplasts, endosperm amyloplasts, or tapetal elaioplasts [23,38–44]. The findings in the present study showed that enzymes involved in carotenoids biosynthesis are likely localized in chloroplasts. The maize ZDS was predicted to localized in chloroplasts by the online TargetP 1.1 Server [45] and carry a 36-amino acid chloroplast transit peptide predicted by the online ChloroP 1.1 Server [46]. A ZDS-YFP fusion protein was transiently expressed in maize protoplasts and tobacco leaves and confirmed the chloroplast localization of the maize ZDS protein (Fig 7 and S6 Fig).

The mutation of the *ZDS* gene in *vp-wl2* blocked the synthesis of downstream carotenoid products, such as lutein, zeaxanthin, β-cryptoxanthin, β-carotene, and α-carotene (Fig 2), some of which are the precursors of ABA. In the 1950s, Robertson [6] reported *vp9* as an ABA-deficient mutant, induced by X-ray exposure. This mutant shows a viviparous phenotype, white or pale yellow endosperm, albino or pale yellow seedlings, and inhibited carotenoid synthesis. The reduction of ABA synthesis in the *vp9* mutant reflects a block in the carotenoid pathway at the third desaturation step, resulting in the accumulation of zeta-carotene [47]. Carotenoid deficiency will also result in albino seedlings and reduced chlorophyll [47,48]. The *vp-wl2* has a similar phenotype as the *vp9* mutant, with white viviparous kernels, albino seedlings, and faint blue-green seedlings observed under low light conditions. The *vp9* mutant is an ABA-deficient mutant, as the ABA content of normal embryos is 2.7-fold higher than that of mutant embryos [49], and another finding showed that the wild type endosperm ABA content was 5.9-fold higher than that of mutant embryos, but the embryos showed a 13.6-fold change [47]. In the present study, the 15 DAP endosperm and embryos were dissected to measure the ABA content, and the normal embryo ABA content was 5.2 to 5.8-fold higher than that of *vp-wl2* mutants, whereas the ABA content was slightly reduced in *vp-wl2* mutant endosperm and showed no significant difference from normal endosperm. Throughout kernel development, a low level of ABA cannot maintain the dormant status of the embryo, leading to vivipary on immature ears.

The block in the third desaturation step of the carotenoid biosynthetic pathway leads to the accumulation of zeta-carotene, indicating that the zeta-carotene desaturase gene is likely the causal gene of the *vp9* locus. However, the *y8* and *y9* mutants also accumulate zeta-carotene. Luo and Wurtzel isolated the maize *ZDS* gene from the endosperm cDNA library in 1999 [22], and further studies showed that *ZDS* was mapped to maize chromosome 7S. The *vp9* locus has been suggested as the candidate locus for the structural gene, and the *y9* locus has been ruled out [23]. Notably, it is hard to confirm the relationship between the *vp9* locus and the *ZDS* gene. The *vp-wl2* is a viviparous mutant, and we cloned *ZDS* as the causal gene of this mutant (gene model ID: GRMZM2G454952). We performed allelic tests between *vp-wl2* and *vp9*; the results showed that these loci are alleles (Table 2). Thus, *ZDS* is the structural gene of the *vp9* locus. To further confirm that *ZDS* is the causal gene of the *vp9* locus, we identified the sequences of *ZDS* gene of three *vp9* mutants. For each allele, the mutant and wild-type *ZDS* gene were cloned, and the results showed that vp9-R and vp9-8113 are frameshift mutants. Compared with their wild-type counterparts, the expression levels of *ZDS* gene is reduced in mutants; the transcript of *ZDS* cannot be detected in the vp9-99-2226-1 mutant, and a large fragment of *ZDS* disappeared in the mutant genome (Figs 4 and 5). The allelic test results and molecular characterization of three *vp9* mutants indicated that the *ZDS* gene (GRMZM2G454952) is the structural gene of the *Vp9* locus.

## Conclusions

In conclusion, *vp-wl2* is a novel allele of *vp9*, and *ZDS* is the causal gene of *vp-wl2* and *vp9*. Mutation of the *ZDS* gene blocks the synthesis of carotenoids, resulting in an ABA deficiency, which eventually leads to vivipary on immature ears.

## Accession number

The sequence data have been deposited in GenBank under the accession numbers KR061340-KR061345.

## Supporting information

S1 Table All the primers used in this study. (XLSX)

S2 Table Segregation analysis of the heterozygous ears in the *vp-wl2* population by fitness test. (XLSX)

S1 Fig Seedlings of some white but dormant kernels of a *vp-wl2*mutant. (TIF)

S2 Fig A fresh segregated ear of *vp-wl2*. (TIF)

S3 Fig Two sequences obtained by DLA method. (PDF)

S4 Fig The abnormal transcripts of the *vp-wl2* mutant. (PDF)

S5 Fig Carotenoids biosynthetic pathway. (PDF)

S6 Fig Subcellular localization of ZDS protein in tobacco. The fused ZDS-YFP protein was transiently expressed in tobacco leaf, bar = 20 μm. Some chloroplasts were marked with white arrows.
(TIF)
