# Supplementary figures and images for: Mutations in the maize zeta-carotene desaturase gene lead to viviparous kernel
Source: PLoS One. 2017 Mar 24;12(3):e0174270. doi: 10.1371/journal.pone.0174270 (PMC5365113; doi:10.1371/journal.pone.0174270)

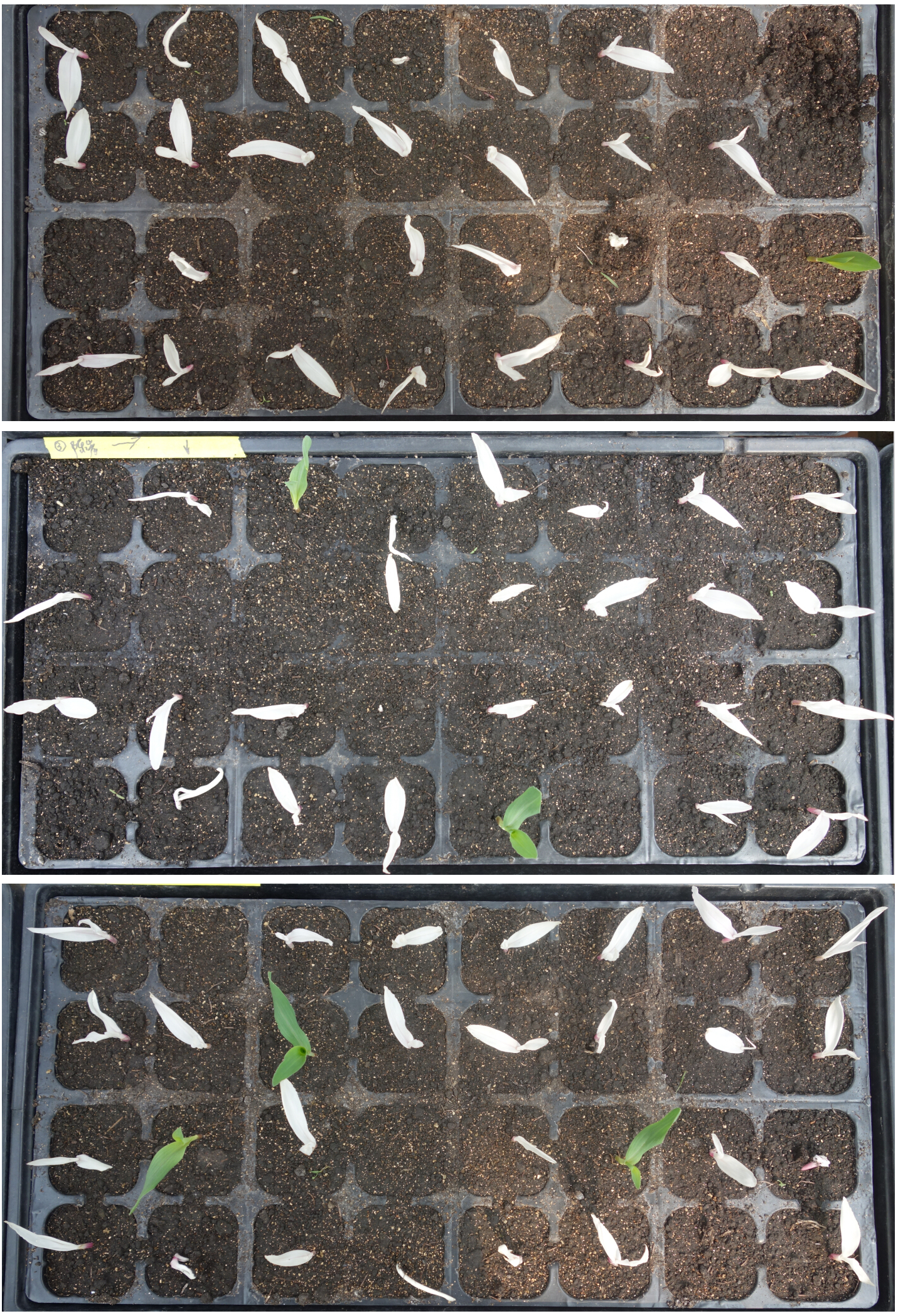

Supplement: S1 Fig — (TIF) [file pone.0174270.s003.tif]

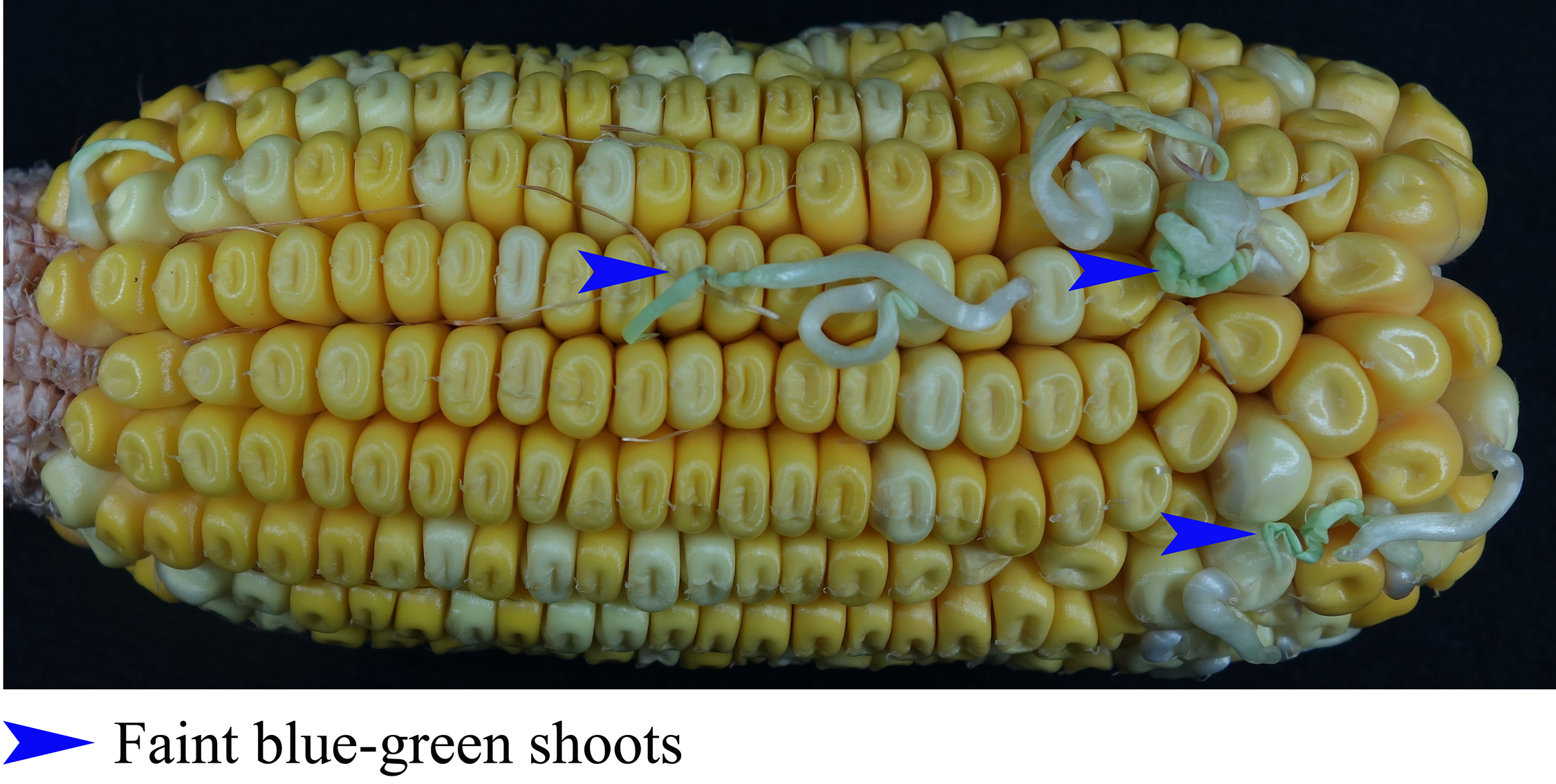

Supplement: S2 Fig — (TIF) [file pone.0174270.s004.tif]

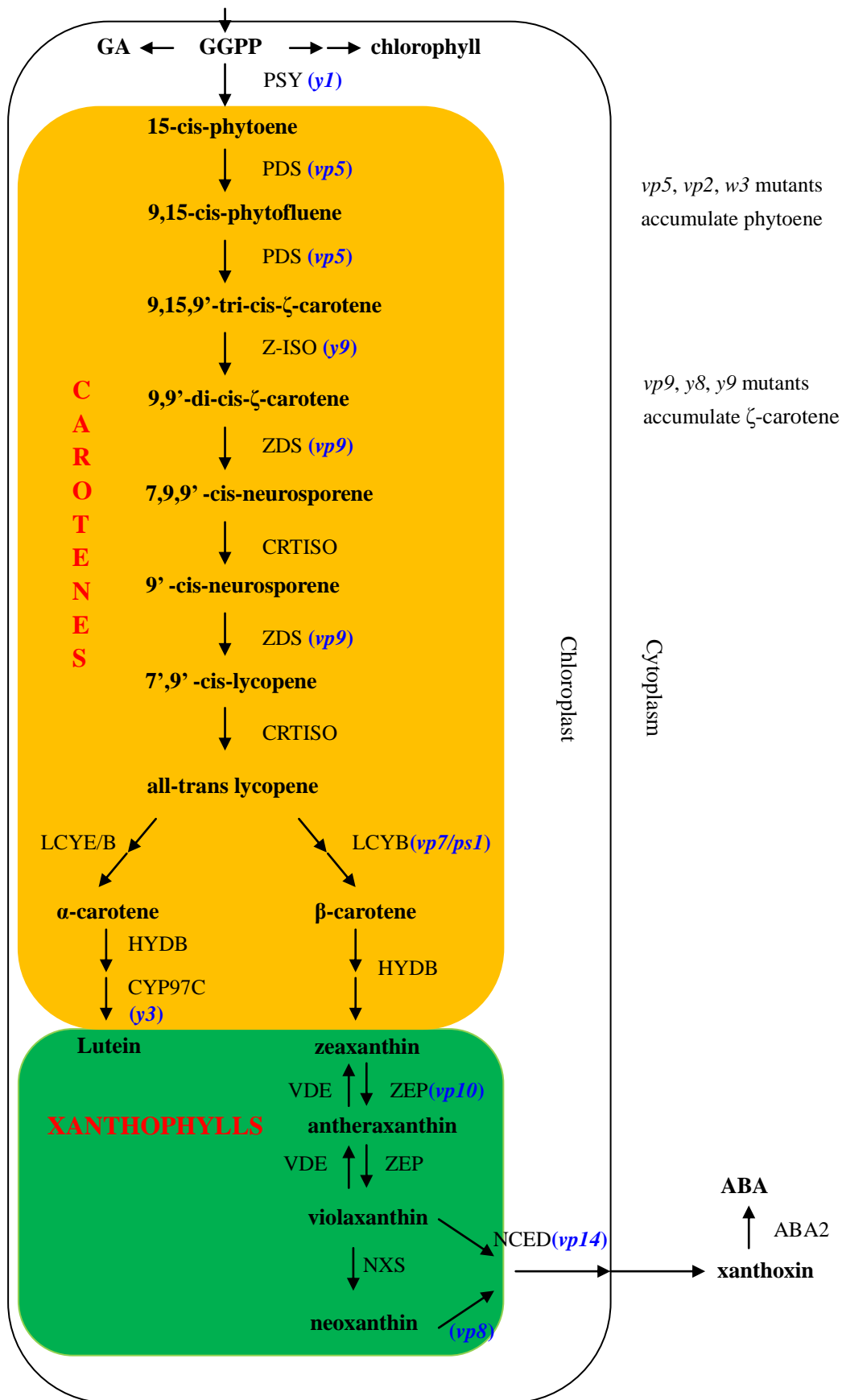

Supplement: S5 Fig — (PDF) [file pone.0174270.s007.pdf]

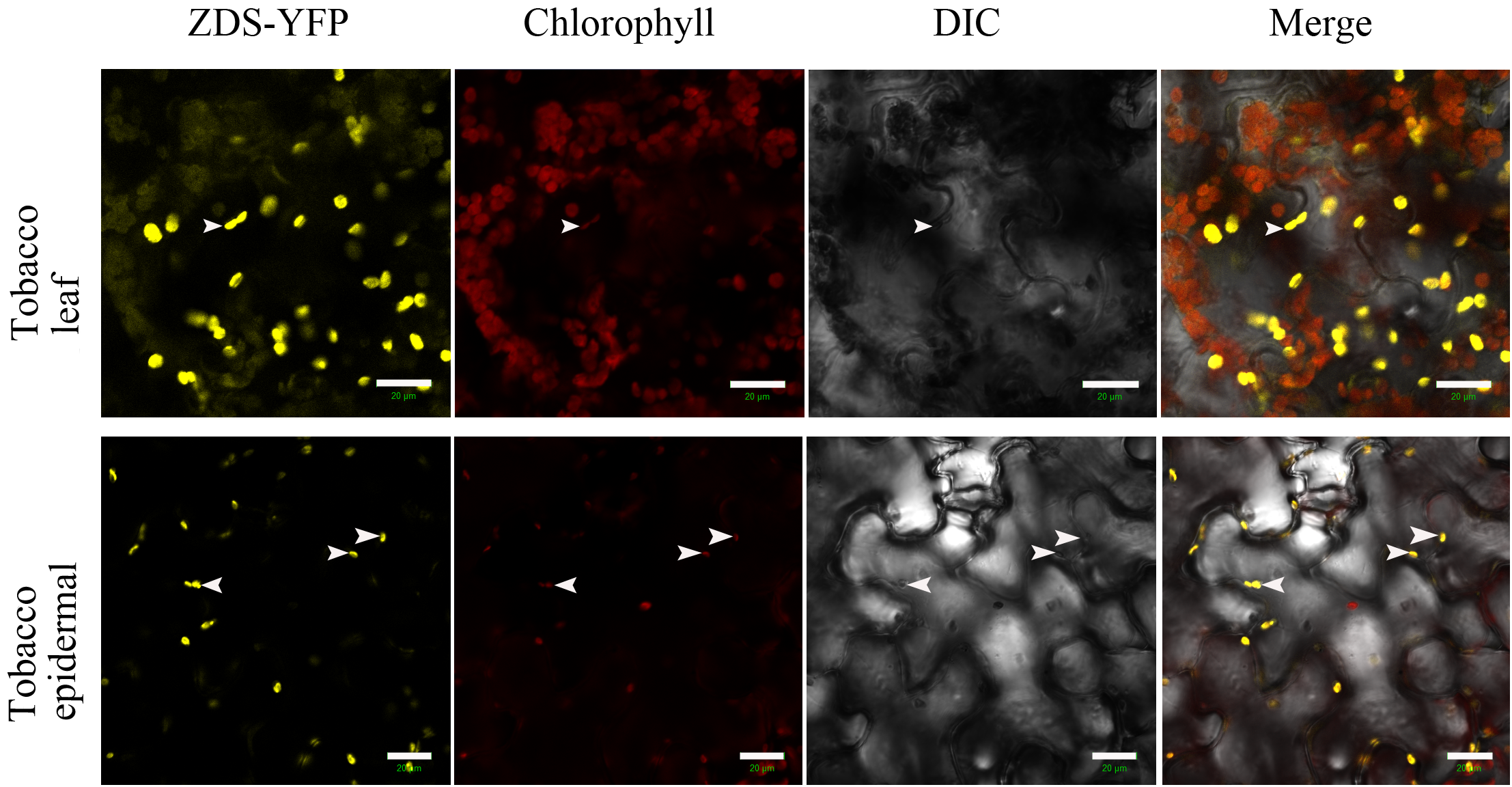

Supplement: S6 Fig — The fused ZDS-YFP protein was transiently expressed in tobacco leaf, bar = 20 μm. Some chloroplasts were marked with white arrows. (TIF) [file pone.0174270.s008.tif]
