# Supplementary material for: Mutations in the maize zeta-carotene desaturase gene lead to viviparous kernel
Source: PLoS One. 2017 Mar 24;12(3):e0174270. doi: 10.1371/journal.pone.0174270 (PMC5365113; doi:10.1371/journal.pone.0174270)
Supplement: S3 Fig — (PDF) [file pone.0174270.s005.pdf]

Mu53s 6R   GCCTCCATTTCGTCGAATCCCTTCCGCTCTTCGTCTATAATGGCAATTATCTCGCTCCCA
Mu53s 22R   GCCTCCATTTCGTCGAATCCCTTCCGCTCTTCGTCTATAATGGCAATTATCTCGCTCCCA
                ****

Mu53s 6R   AAGGGTTATTCCCACCCGAGCCAGAGCACTACAGGGGCCCGAAGCTCAAGGTGGCCATCA
Mu53s 22R   AAGGGTTATTCCCACCCGAGCCAGAGCACTACAGGGGCCCGAAGCTCAAGGTGGCCATCA
                ****

Mu53s 6R   TAGGGGCAGGCCTTGCGGGCATGACATGCTGTGA
Mu53s 22R   TAGGGGCAGGCCTTGCGGACATGACATGCTGTGA
                ****************** ***************

> Mu53s 6R
GCCTCCATTTCGTCGAATCCCTTCCGCTCTTCGTCTATAATGGCAATTATCTCGCTCCCAAAGGGTTATTCCCACCCGAGCCA
GAGCACTACAGGGGCCCGAAGCTCAAGGTGGCCATCATAGGGGCAGGCCTTGCGGGCATGACATGCTGTGA
> Mu53s 22R
GCCTCCATTTCGTCGAATCCCTTCCGCTCTTCGTCTATAATGGCAATTATCTCGCTCCCAAAGGGTTATTCCCACCCGAGCCA
GAGCACTACAGGGGCCCGAAGCTCAAGGTGGCCATCATAGGGGCAGGCCTTGCGGACATGACATGCTGTGA
