# Supplementary material for: Mutations in the maize zeta-carotene desaturase gene lead to viviparous kernel
Source: PLoS One. 2017 Mar 24;12(3):e0174270. doi: 10.1371/journal.pone.0174270 (PMC5365113; doi:10.1371/journal.pone.0174270)
Supplement: S4 Fig — (PDF) [file pone.0174270.s006.pdf]

Note: Yellow backgroud represents the CDS sequences of *ZDS* gene; green background represents the partial sequences of *Mu9*; red background represents the intron sequences of *ZDS* gene.

Transcript 1 of *vp-wl2* mutant

AACGCTGCCGACCACGGCTGCCGCCAACCACCCGCCCCACCGCTGCCGAGTGCTAGCCATTTGGAGCTGCC
GCGCCATGGCGTCCGTGGCCGCCACCACCACGCTGGCACCGGCACTCGCGCCCCGGCGCCGGGCGCGGCC
AGGGAGCGGGCTGCTGCCGCCGCGCCGGGCCTCGGCCGTCCGCTGCTCGCTCGACAGTAACGTCTCCGAC
ATGGGCGTCAACGCTCCCAAAGAGATAATTGCCATTATAGACGAAGAGCGGAAGGGATTCGACGAAATGGA
GGCCATGGCGTTGGCTTCTATGATCTGGAGACGCAGAGGACAGCCAATCGCCAAAACAGAAAGGTGACAG
CGCTTGGAGCTCCTTAAACAGTTGGCGTACTCCTCTCCTCGCCGAATTGGAGTGTTCTCGGGAGCTGGCGTC
TTCCTACTGCGGCTGCTTCCGGTTTCCTGTTCGTTTTCTCTGATTTTGGATCCATGGACTTGACGCCCAGTTTC
AATTCGCTAGACTCCAACGGCATTCCCAACTCCCCCGATGTAGATCCGGCATTGGGCGAAACAGGTGGCAGT
GAGGTTGATTTGTACGAGTCCCGTCCGTTTATCGGTGGTAAGGTTGGCTCCTTTGTTGACAGGCAAGGAAA
CCATATCGAGATGGGGCTGCATGTGTTCTTCGGGTGCTACAGCAATCTCTTCCGCCTCATGAAGAAGGTTGG
CGCTGATAATAATCTGCTGGTGAAGGAACATACCCATACTTTTGTAAATAAAGGGGGCACGATTGGTGAACTT
GATTTTCGGTTCCCGGTGGGAGCTCCGTTACATGGCATTCAAGCATTCCTAAGAACTAATCAGCTCAAGGTTT
ATGATAAAGCAAGAAATGCAGTTGCTCTTGCCCTTAGTCCAGTTGTTCGGGCTCTGGTTGATCCTGATGGTG
CATTGCAGCAAGTGCGGGACTTGGATGATATAAGTTTCAGTGATTGGTTCATGTCCAAAGGGGGTACTCGG
GAGAGTATCACAAGAATGTGGGATCCTGTTGCTTACGCTTTGGGTTTCATTGACTGTGATAATATCAGTGCAC
GTTGCATGCTTACTATTTTCACCTTGTTTGCCACAAAGACAGAGGCATCCCTGTTACGCATGTTAAAGGGTTC
ACCTGATGTTTACTTAAGTGGTCCAATAAAGAAGTATATAACAGACAGGGGTGGTAGGTTTCACTTAAGGTG
GGGATGCAGAGAGGTTCTCTATGAGAAATCACCTGATGGAGAGACCTATGTTAAGGGCCTTCTACTCACCAA
GGCTACAAGTAGAGAGATAATCAAAGCTGATGCATACGTCGCAGCCTGTGATGTTCCGGGTATCAAAGATTA
CTTCCATCAGAATGGAGGGAATGGGAAATGTTTGACAATATCTACAAGTTAGATGGTGTCCCTGTTGTCACTG
TCCAGCTCCGCTACAACGGATGGGTCACTGAACTTCAAGATTTGGAGAAATCAAGACAACTGCAAAGGGCG
GTTGGGTTGGATAACCTTTTGTACACGGCGGATGCAGACTTTTCCTGTTTTTCGGACCTTGCTCTCTCATCTC
CTGCTGATTACTACATTGAAGGGCAAGGTTCCCTGATCCAAGCTGTGCTGACTCCTGGAGATCCATACATGCC
ATTGCCAAACGAGGAGATCATTAGTAAGGTTCAAAAGCAGGTTGTAGAACTGTTCCCATCTTCCCGGGGCTT
GGAAGTTACATGGTCCAGTGTGGTAAAGATCGGACAATCGCTGTACCGTGAGGCTCCTGGAAACGACCCAT
TCAGGCCTGATCAGAAGACGCCCGTTAAAAACTTCTTCCTCTCTGGATCTTACACGAAACAGGACTACATCG
ACAGCATGGAAGGAGCAACTCTCTCCGGCAGGCGAACGTCGGCCTACATCTGCGGTGCCGGGGAGGAGCT
GCTGGCCCTCCGAAAGAAGCTACTCATCGACGACGGCGAGAAGGCGCTGGGGAACGTTCAAGTCCTGCAG
GCTAGCTGAACAACCCCTCCTGCACTGCAGAGAAGCTTGGATCTTTCCAACCACACATACATGCTGGAATGG
ACAAACCAACCAACCATTGTCTTTTCTCGCTTCAGGGTGCTGGCGATTCCCGCAGCGACCTCCTGTGTATCGT
ATCCAATTTGAGCA

Transcript 2 of *vp-wl2* mutant

CCCGCCCCACCGCTGCCGAGTGCTAGCCATTTGGAGCTGCCGCGCCATGGCGTCCGTGGCCGCCACCACCA
CGCTGGCACCGGCACTCGCGCCCCGGCGCCGGGCGCGGCCAGGGAGCGGGCTGCTGCCGCCGCGCCGG
GCCTCGGCCGTCCGCTGCTCGCTCGACAGTAACGTCTCCGACATGGGCGTCAACGCTCCCAAAGAGATAATT
GCCATTATAGACGAAGAGCGGAAGGGATTCGACGAAATGGAGGCCATGGCGTTGGCTTCTATGATCTGGAG
ACGCAGAGGACAGCCAATCGCCAAAACAGAAAGGTGACAGCGCTTGGAGCTCCTTAAACAGGTTGATTTG
TACGAGTCCCGTCCGTTTATCGGTGGTAAGGTTGGCTCCTTTGTTGACAGGCAAGGAAACCATATCGAGATG
GGGCTGCATGTGTTCTTCGGGTGCTACAGCAATCTCTTCCGCCTCATGAAGAAGGTTGGCGCTGATAATAAT
CTGCTGGTGAAGGAACATACCCATACTTTTGTAAATAAAGGGGGCACGATTGGTGAACTTGATTTTCGGTTC
CCGGTGGGAGCTCCGTTACATGGCATTCAAGCATTCCTAAGAACTAATCAGCTCAAGGTTTATGATAAAGCA
AGAAATGCAGTTGCTCTTGCCCTTAGTCCAGTTGTTCGGGCTCTGGTTGATCCTGATGGTGCATTGCAGCAA
GTGCGGGACTTGGATGATGTAAGTATCCCTTGCTTTTATTTCAGATCCATTAAGAGTGTGCATCAGGAGCATT
CATTTGCAATAGTCAGTATCTTTTCAAAATGTGTTTTGTTCTAACTCGCTGATAACTAAACTTTTTTGTCAATTT
TTTTCTCAAACACTCGTTAATTTTATTTTAACCCATTTCAGATAAGTTTCAGTGATTGGTTCATGTCCAAAGGG
GGTACTCGGGAGAGTATCACAAGAATGTGGGATCCTGTTGCTTACGCTTTGGGTTTCATTGACTGTGATAATA
TCAGTGCACGTTGCATGCTTACTATTTTCACCTTGTTTGCCACAAAGACAGAGGCATCCCTGTTACGCATGTT
AAAGGGTTCACCTGATGTTTACTTAAGTGGTCCAATAAAGAAGTATATAACAGACAGGGGTGGTAGGTTTCA
CTTAAGGTGGGGATGCAGAGAGGTTCTCTATGAGAAATCACCTGATGGAGAGACCTATGTTAAGGGCCTTCT
ACTCACCAAGGCTACAAGTAGAGAGATAATCAAAGCTGATGCATACGTCGCAGGTTTTTTTCCTCTGTCTTGT
CTTTCTTCTTTTACTAGCATGTCCAACCATACACACTTAACTTGGTGTTCCACAGCCTGTGATGTTCCGGGTAT
CAAAAGATTACTTCCATCAGAATGGAGGGAATGGGAAATGTTTGACAATATCTACAAGTTAGATGGTGTCCC
TGTTGTCACTGTCCAGCTCCGCTACAACGGATGGGTCACTGAACTTCAAGATTTGGAGAAATCAAGACAACT
GCAAAGGGCGGTTGGGTTGGATAACCTTTTGTACACGGCGGATGCAGACTTTTCCTGTTTTTCGGACCTTG
CTCTCTCATCTCCTGCTGATTACTACATTGAAGGGCAAGGTTCCCTGATCCAAGCTGTGCTGACTCCTGGAGA
TCCATACATGCCATTGCCAAACGAGGAGATCATTAGTAAGGTTCAAAAGCAGGTTGTAGAACTGTTCCCATCT
TCCCGGGGCTTGGAAGTTACATGGTCCAGTGTGGTAAAGATCGGACAATCGCTGTACCGTGAGGCTCCTGG
AAACGACCCATTCAGGCCTGATCAGAAGACGCCCGTTAAAAACTTCTTCCTCTCTGGATCTTACACGAAACA
GGACTACATCGACAGCATGGAAGGAGCAACTCTCTCCGGCAGGCGAACGTCGGCCTACATCTGCGGTGCCG
GGGAGGAGCTGCTGGCCCTCCGAAAGAAGCTACTCATCGACGACGGCGAGAAGGCGCTGGGGAACGTTC
AAGTCCTGCAGGCTAGCTGAACAACCCCTCCTGCACTGCAGAGAAGCTTGGATCTTTCCAACCACACATACA
TGCTGGAATGGACAAACCAACCAACCATTGTCTTTTCTCGCTTCAGGGTGCTGGCGATTCCCGCAGCGACCT
CCTGTGTATCGTATCCAATTTGAGCA

Larger but blurred band amplified by primers ZUTR-3F and ZUTR-3R

GTCTCCGACATGGGCGTCAACGCTCCCAAAGAGATAATTGCCATTATAGACGAAGAGCGGAAGGGATTCGA
CGAAATGGAGGCCATGGCGTTGGCTTCTATGATCTGGAGACGCAGAGGACAGCCAATCGCCAAAACAGAA
AGGTGACAGCGCTTGGAGCTCCTTAAACAGTTGGCGTACTCCTCTCCTCGCCGAATTGGAGTGTTCTCGGG
AGCTGGCGTCTTCCTACTGCGGCTGCTTCCGGTTTCCTGTTCGTTTTCTCTGATTTTGGATCCATGGACTTGA
CGCCCAGTTTCAATTCGCTAGACTCCAACGGCATTCCCAACTCCCCCGATGTAGATCCGGCATTGGGCGAAA
CAGGTGGCAGTGAGGTTGATTTGTACGAGTCCCGT
